# FSTL1-USP10-Notch1 Signaling Axis Protects Against Cardiac Dysfunction Through Inhibition of Myocardial Fibrosis in Diabetic Mice

**DOI:** 10.3389/fcell.2021.757068

**Published:** 2021-12-09

**Authors:** Linhe Lu, Jipeng Ma, Yang Liu, Yalan Shao, Xiang Xiong, Weixun Duan, Erhe Gao, Qianli Yang, Shasha Chen, Jian Yang, Jun Ren, Qijun Zheng, Jincheng Liu

**Affiliations:** ^1^ Department of Cardiovascular Surgery, Xijing Hospital, The Air Force Medical University, Xi’an, China; ^2^ Center for Translational Medicine, Lewis Katz School of Medicine at Temple University, Philadelphia, PA, United States; ^3^ Department of Ultrasound, Xijing Hospital, The Air Force Medical University, Xi’an, China; ^4^ Department of Cardiology and Shanghai Institute of Cardiovascular Diseases, Zhongshan Hospital Fudan University, Shanghai, China; ^5^ Department of Clinical Medicine and Pathology, University of Washington, Seattle, WA, United States; ^6^ Department of Cardiovascular Surgery, Shenzhen People’s Hospital, The Second Clinical Medical College, Jinan University, Shenzhen, China

**Keywords:** fibrosis, follistatin-like protein 1, ubiquitin-specific protease 10, apoptosis, diabetes cardiomyopathy, myocardial infarction

## Abstract

The incidence of type 2 diabetes mellitus (T2DM) has been increasing globally, and T2DM patients are at an increased risk of major cardiac events such as myocardial infarction (MI). Nevertheless, the molecular mechanisms underlying MI injury in T2DM remain elusive. Ubiquitin-specific protease 10 (USP10) functions as a NICD1 (Notch1 receptor) deubiquitinase that fine-tunes the essential myocardial fibrosis regulator Notch signaling. Follistatin-like protein 1 (FSTL1) is a cardiokine with proven benefits in multiple pathological processes including cardiac fibrosis and insulin resistance. This study was designed to examine the roles of FSTL1/USP10/Notch1 signaling in MI-induced cardiac dysfunction in T2DM. High-fat-diet-treated, 8-week-old C57BL/6J mice and *db/db* T2DM mice were used. Intracardiac delivery of AAV9-FSTL1 was performed in T2DM mice following MI surgery with or without intraperitoneal injection of crenigacestat (LY3039478) and spautin-1. Our results demonstrated that FSTL1 improved cardiac function following MI under T2DM by reducing serum lactate dehydrogenase (LDH) and myocardial apoptosis as well as cardiac fibrosis. Further *in vivo* studies revealed that the protective role of FSTL1 against MI injury in T2DM was mediated by the activation of USP10/Notch1. FSTL1 protected cardiac fibroblasts (CFs) against DM-MI-induced cardiofibroblasts injury by suppressing the levels of fibrosis markers, and reducing LDH and MDA concentrations in a USP10/Notch1-dependent manner. In conclusion, FSTL1 treatment ameliorated cardiac dysfunction in MI with co-existent T2DM, possibly through inhibition of myocardial fibrosis and apoptosis by upregulating USP10/Notch1 signaling. This finding suggests the clinical relevance and therapeutic potential of FSTL1 in T2DM-associated MI and other cardiovascular diseases.

## Introduction

The prevalence of diabetes mellitus (DM) worldwide is increasing, and it is estimated to reach 693 million patients by 2045. Diabetes poses a major health care burden with respect to cost and health outcomes of patients with DM (Cole & Florez 2020; Ritchie & Abel 2020). 90–95% of diabetes cases are type 2 diabetes mellitus (Kenny & Abel 2019). Although diet and exercise represent a practical avenue for the treatment and prevention of type 2 diabetes mellitus (T2DM) (Magkos et al., 2020; Reynolds et al., 2020), coronary ischemic heart diseases are considered the main causes of heart failure and mortality in patients with DM (Del Re et al., 2019; Dillmann 2019). Myocardial infarction (MI) is one of the major cardiac sequelae in patients with DM, accompanied by mitochondrial dysfunction, inflammation, apoptosis, and myocardial fibrosis (Birnbaum et al., 2019; Lu et al., 2020a; Frangogiannis 2020; Wu et al., 2021). Notably, myocardial fibrosis is an important pathophysiological process in the etiology of heart failure in DM patients with myocardial infarction, although the underlying mechanism remains elusive.

Follistatin-like protein 1 (FSTL1) is a secreted cardiokine glycoprotein that belongs to the follistatin family, the levels of which are upregulated in the stressed heart (Ogura et al., 2012; Maruyama et al., 2016). Previous studies reported a protective role of FSTL1 in doxorubicin-induced cardiotoxicity through upregulation of Nrf2 to suppress apoptosis and oxidative stress (Zhao et al., 2020). FSTL1 insufficiency leads to venous wall and atrial fibrosis by switching on SMAD3 signaling (Jiang et al., 2020). Further, Wei et al. applied an epicardial patch with human FSTL1 protein to rescue cardiac dysfunction in swine and mouse MI models through inhibition of myocardial fibrosis (Wei et al., 2015; Shen et al., 2019; Hu et al., 2020; Xi et al., 2020). FSTL1 was also implicated in the regulation of insulin resistance and circulating FSTL1 levels were elevated in patients with T2DM (Xu et al., 2020). However, the role of FSTL1 in MI-induced cardiac remodeling in the setting of T2DM has not been elucidated.

Ubiquitin-specific protease 10 (USP10) is a deubiquitinase that catalyzes the hydrolysis reaction and removes conjugated ubiquitin from its target proteins. In response to cell stress, USP10 was translocated from the cytoplasm to the nucleus to regulate cell cycle and apoptosis (Lim et al., 2019; Zhang et al., 2020). Previous studies demonstrated that USP10 protected cells against pathological injury in numerous organs (Yuan et al., 2010; Deng et al., 2016; Wang et al., 2020). It was reported that USP10 displayed the vital role in premature senescence of cardiac progenitor cells by regulation of p53 and p21 proteins (Cai et al., 2016). Inhibition of USP10 exacerbated cardiac hypertrophy by regulating sirtuin 6 (Sirt6) signaling (Zhang et al., 2020). In the endothelium, USP10 functioned as a NICD1 deubiquitinase that fine-tuned endothelial Notch signaling responses during angiogenic sprouting (Lim et al., 2019). Our earlier report revealed that upregulation of Notch1 ligand Jagged-1 and its intracellular domain decreased transverse aortic constriction-induced myocardial fibrosis, which could be reversed by Notch inhibition (Chen X. et al., 2019). However, the pathological role of Notch1/USP10 signaling in MI-induced cardiac remodeling and fibrosis in T2DM remains largely unexplored. The present study employed both *in vivo* and *in vitro* experiments to decipher the changes in Notch1 and USP10 and to evaluate the possible interaction between FSTL1 and USP10 in the pathological mechanism of MI-induced myocardial fibrosis in T2DM.

## Materials and Methods

### Ethics Statement

All experimental animals (C57BL/6J, male, 8-week-old) involved in the present study were provided by the Laboratory Animal Center of the Air Force Medical University. All experimental procedures were performed in compliance with the 2011 Guide for the Care and Use of Laboratory Animals, and the study protocol was approved by the Air Force Military Medical University Experimental Animal Research Committee (Xi’an, China). Before the experiment, mice were maintained under 12:12 h dark-and-light cycles for 1 week, at 25°C.

### Reagents

Recombinant human Fstl1 was purchased from PeproTech Co. (New Jersey). The plasmids harboring the expressing cassette of USP10 and the control vector were obtained from VigeneBio (Shandong, China). Crenigacestat (LY3039478) was purchased from Selleckchem Chemicals (Houston, TX). The terminal deoxynucleotidyl transferase-mediated dUTP nick end labeling (TUNEL) assay kit was obtained from Roche (Mannheim, Germany). DAPI (4′,6-Diamino-2-phenylindole) was purchased from Sigma-Aldrich (St. Louis, MO, United States). Anti-alpha smooth muscle actin antibody (α-SMA, ab7817), Collagen I (ab260043), MMP9 (ab76003), USP10 (ab70895), Notch1 (ab52627), and FSTL1 (ab223287) were purchased from Abcam (London, United Kingdom). Rabbit polyclonal antibody DIP2A (CA, United States) was obtained from GeneTex, and α-tubulin was purchased from CST (MA, United States), Goat anti mouse (ZB-2305), goat anti-rabbit (ZB-2301), and rabbit anti-goat (ZB-2306) secondary antibodies were purchased from the ZSGB-Bio (Beijing, China).

### Establishment of T2DM Using High-Fat Diet Intake

HFD (containing 60 kcal% fat, 20 kcal% carbohydrate and 20 kcal% protein) and the normal chow diet (containing 10% kcal fat, 20% kcal protein, and 70% kcal carbohydrate) were obtained from Research Diets (Inc, NJ, United States). To mimic T2DM in humans, mice were fed HFD for 4 weeks, and then injected intraperitoneally with 100 μL of 150 mg/kg streptozotocin (STZ, S0130, Sigma-Aldrich, MO, United States), following another HFD feeding for 8 weeks. Mice fed with the standard normal diet (ND) were administered intraperitoneal injection with an equal volume of 0.1 M citrate buffer. All mice were fasted overnight before STZ injection. Mice were deemed to have DM with fasting plasma glucose (FPG) of >11.1 mmol/L at 3 days’ post injection. Otherwise, mice were excluded from the experiment. Before being sacrificed, all mice were continuously fed with either the HFD or ND for 4 weeks post the MI/Sham protocol (Lu et al., 2020b; Yu et al., 2021).

### Protocol for MI Injury and Intracardiac Injection

MI surgery was performed as described previously (Lu et al., 2020a). An isoflurane delivery system was employed to anesthetize the mice with a 1.5–2% isoflurane-oxygen mixture in the surgical plane. The body temperature of mice was maintained using a heating pad. The left chest was cut to create a tiny incision in the fourth intercostal space. The heart was exteriorized from the thoracic cavity and the left anterior descending coronary artery was ligated using a 6-0 silk suture. After ligation, the heart was replaced into the intrathoracic cavity. The same surgery was performed for the sham-operated group without ligation of the left coronary artery. For expression of cardiac-specific FSTL1 in mice, adeno-associated virus (AAV) serotype 9 (AAV9)-FSTL1 and AAV9-NC (negative control) virus (Hanbio biotechnology Co., Ltd, Shanghai, China) were administered into the left ventricle using three evenly spaced injections (20 μL each, concentration: 5×10^10^ vg/mL) 8 days before the operation.

### *In vivo* Experimental Procedure

Mice were randomly assigned to the groups below in the first experiment (*n* = 10–15 per group). (I) Sham group (Sham): mice were fed the standard ND and underwent a sham operation. (II) T2DM group (T2DM): mice were fed the HFD and underwent a sham operation. (III) T2DM with MI group (T2DM-MI): mice were fed the HFD and underwent MI surgery. (IV) *db/db* group (*db/db*): mice were fed the standard ND and underwent a sham operation. (V) *db/db* with MI group (*db/db*-MI): *db/db* mice were fed the standard ND and underwent MI surgery.

In the second experiment, mice were randomly divided into four groups (*n* = 20–25 per group). (I) Sham group (Sham): mice underwent a sham operation and were administered an intracardiac injection (AAV9-NC, 20 μL). (II) FSTL1 group (FSTL1): mice underwent a sham operation and were administered an intracardiac injection (AAV9-FSTL1, 20 μL). (III) T2DM with MI surgery group (T2DM-MI, T2MI): HFD-induced T2DM mice underwent MI surgery and were administered intracardiac injection (AAV9-NC, 20 μL). (IV) T2DM with MI surgery and FSTL1 treatment group (T2DM-MI-AAV9-FSTL1, T2MI-FSTL1): HFD-induced T2DM mice underwent MI surgery and were administered intracardiac injection (AAV9-FSTL1, 20 μL).

In the third experiment, HFD-induced T2DM mice were randomly divided into the following groups (*n* = 20–25 per group). (I) T2DM mice with MI surgery group (T2DM-MI, T2MI): HFD-induced T2DM mice were administered an intracardiac injection (AAV9-NC, 20 μL) post-MI, and treated with 20 μL intraperitoneal saline (once every other day for 4 weeks). (II) FSTL1 treated group (T2DM-MI-FSTL1, T2MI-FSTL1): HFD-induced T2DM mice were administered an intracardiac injection (AAV9-FSTL1, 20 μL) post-MI, and were treated with 20 μL intraperitoneal saline (once every other day for 4 weeks). (III) T2DM-MI-FSTL1-spautin-1 treatment group (T2DM-MI-FSTL1-spautin-1, T2MI-FSTL1-SPA): HFD-induced T2DM mice were administered an intracardiac injection (AAV9-FSTL1, 20 μL) post-MI, and injected intraperitoneally with 20 μL spautin-1 (20 mg/kg/2 days for 4 weeks) (Liao et al., 2019a). (IV) Spautin-1-inhibited DM mice group (T2DM-MI-Spautin-1, T2MI-SPA): HFD-induced T2DM mice were administered an intracardiac injection (AAV9-NC, 20 μL) post-MI, and treated with 20 μL intraperitoneal spautin-1 (20 mg/kg/2 days for 4 weeks).

### Echocardiography

Mice were anesthetized with 2–3% isoflurane and maintained with 1–1.5% isoflurane-oxygen mixture. Cardiac function was examined using a Vevo 2100 high-resolution imaging system (Visual Sonics, Toronto, ON, Canada) as previously described (Lu et al., 2020a; Lu et al., 2020b). Left ventricular ejection fraction (LVEF), left ventricular fractional shortening (LVFS), left ventricular internal diameter at end-diastole (LVIDd), and left ventricular internal diameter at end-systole (LVIDs were assessed using the Vevo Lab Workstation software (V3.1.0).

### Protein Extraction and Western Blot Analysis

At the end of the experiments, left ventricular tissues of myocardial infarction tissue as well as its border zone and mouse cardiac fibroblasts were harvested and washed with ice-cold PBS, and were then lysed using RIPA lysis buffer (Beyotime Biotechnology, Shanghai, China), with a phosphatase inhibitor and a protease inhibitor and maintained on ice for 25 min (Lu et al., 2020a; Lu et al., 2020b). Subsequently, lysates were collected and the protein concentration was determined using a BCA protein assay kit (Thermo Fisher Scientific, Waltham, MA, United States) after being boiled at 100°C for 7 min with 5X loading buffer. Equal amount of each sample was separated by 10–12% SDS-PAGE. The proteins were transferred to a polyvinylidene fluoride (PVDF) membrane and were subsequently probed with first antibodies after blocking with 5% non-fat milk overnight at 4°C. Then the membranes were subsequently incubated with secondary antibodies (ZSGB-Bio, Beijing, China) at 25–28°C for 1.5–2 h. Protein bands were visualized and quantified with Image Lab software (Bio-Rad Laboratories, Hercules, CA, United States).

### Hematoxylin and Eosin and Masson’s Trichrome Staining

Cardiac tissues of the left ventricle were fixed in 4% paraformaldehyde for at least 3 days. The samples were embedded in paraffin and cut into 5-μm thick sections. Sections were separately stained with HE for morphological assessment and with Masson’s trichrome stain to assess myocardial fibrosis. The morphological changes and deposition of collagen were observed by microscopy (Nikon, Tokyo, Japan) with 3–5 randomly selected fields in each group and the images were analyzed by Image-Pro Plus software version 6.0 (Media Cybernetics, Maryland, United States).

### Primary Mouse Cardiac Fibroblasts Isolation and Culture

Male adult C57BL/6J mice were used to isolate cardiac fibroblasts using collagenase-based digestion as previously described (Piccoli et al., 2017). Hearts were harvested and cut into small pieces, then washed with ice-cold ADS buffer containing heparin (20 mM Hepes, 116 mM NaCl, 5 mM KCl, 1.0 mM NaH_2_PO_4_, 5.5 mM glucose, 0.8 mM Mg_2_SO_4_, 40 units/mL heparin; pH 7.4) to remove all plasma contaminants. The pieces were pre-digested in an enzyme solution (81 units/mL collagenase II, 0.1 mg/ml pancreatin in ADS buffer without heparin) at 37°C for 10 min and incubated for 6 × 20 min digestion rounds at 37°C. The fibroblast-containing supernatants were transferred to a new tube, diluted with fetal bovine serum (FBS), and centrifuged for 5 min at 1,000 rpm, The pellet was re-suspended in complete DMEM with 10% FBS before being filtered using a 100 μm cell strainer. Cells were cultured under 5% CO_2_ and 95% air at 37°C and were used in the subsequent experiment at 2–4 passage.

### Cell Viability

**FIGURE 1:**
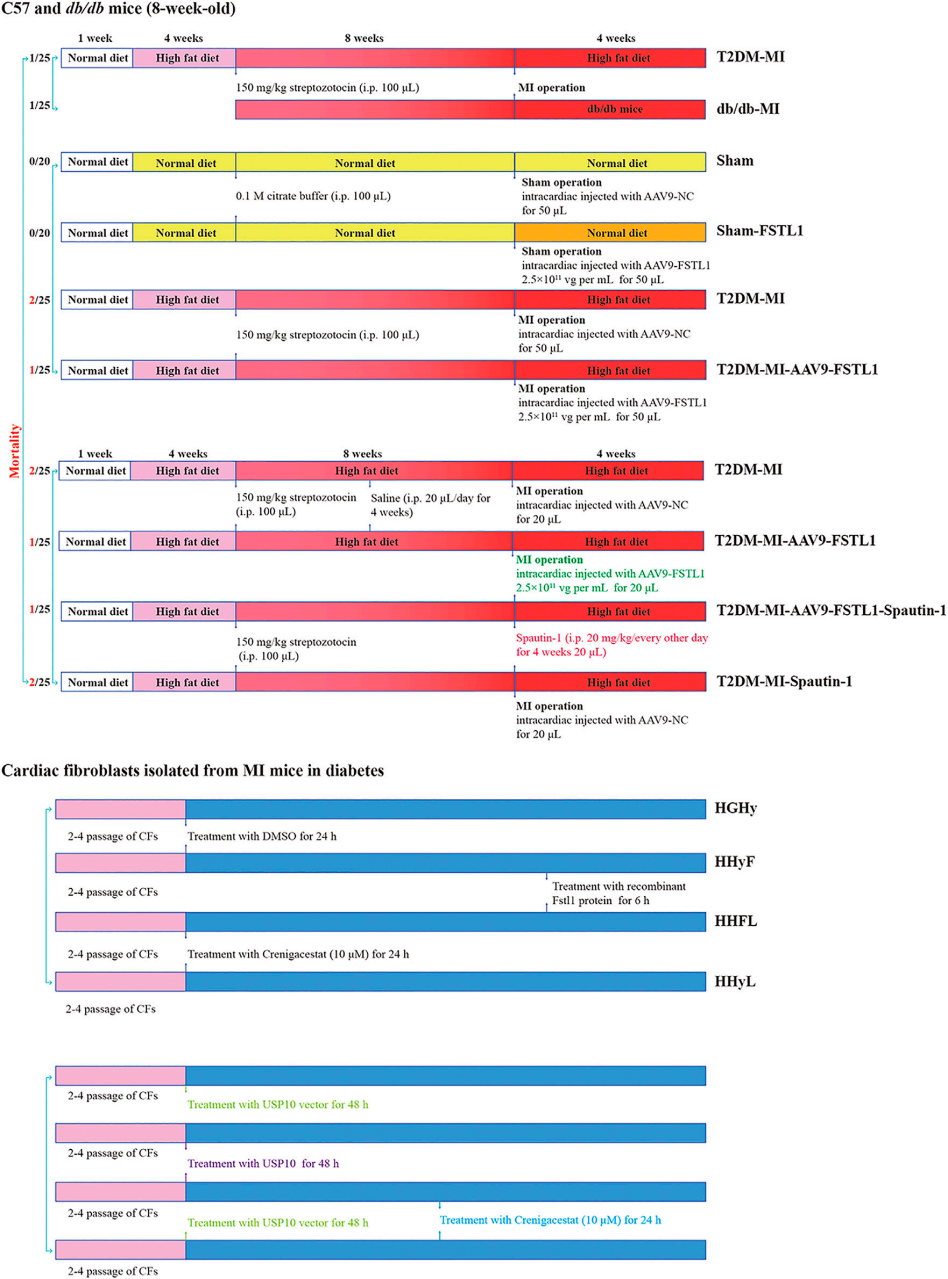
Protocol for *in vivo* and *in vitro* experiments and animal mortality for each group.

Cell viability was detected using the Cell Counting Kit-8 (CCK-8) (Dojindo Kumamoto, Japan) based on manufacturer’s instructions. Cardiac fibroblasts (CFs) underwent various treatments in a 96-well culture plate as shown in Figure 1, with 10 μL of sterile CCK-8 added to each well. Subsequently, this plate was incubated for 2 h at 37°C. Absorbance values were detected using a microplate reader at an excitation wavelength of 450 nm.

### *In vitro* Experiment

Primary mouse CFs were isolated from HFD-induced T2DM mice that underwent MI surgery, and incubated in a complete DMEM (Dulbecco’sModified Eagle Medium) at passage 2–4 and were assigned to the groups below. (I) Fibroblasts that isolated from T2DM-MI (HGHy) group: isolated CFs were treated with dimethyl sulfoxide (DMSO) as control. (II) HHyF group: isolated CFs were treated with 50 ng/ml recombinant Fstl1 protein for 6 h (Maruyama et al., 2016). (III) HHFL group: isolated CFs were treated with crenigacestat (LY3039478, 10 μM) for 18 h before recombinant Fstl1 protein administration, and then co-cultured for 6 h (Mancarella et al., 2020). (IV) HHyL group: isolated CFs were treated with crenigacestat for 24 h. (V) Isolated CFs were treated with USP10 plasmids vector for 48 h (VI) Isolated CFs were treated with USP10 plasmids for 48 h (Liao et al., 2019b). (VII) Isolated CFs were treated with USP10 plasmids for 24 h and then co-cultured with crenigacestat for 24 h (VIII) Isolated CFs were treated with USP10 plasmids vector for 24 h and then co-cultured with crenigacestat for 24 h.

### Immunostaining

CFs were washed with PBS supplemented with penicillin-streptomycin post-experimental. Then CFs were fixation with ice-cold 100% methanol (Fisher) at −20°C. Fixed cells were treated with Triton-X 100 for 15 min and then incubated with mouse anti-alpha smooth muscle actin (α-SMA) (5 μg/ml, A5228, Sigma-Aldrich) overnight at 4°C. After washing with PBST, cells were incubated with Alexa Fluor 488-conjugated donkey anti-mouse secondary antibodies (2 μg/ml, A21206, Invitrogen) and incubated for 1 hour in the dark. Finally, the nuclei of CFs were counterstained using Hoechst 33342 (Thermo, 1 μg/ml) and the images were observed under an Olympus fluorescence FV100i microscope (Olympus, Japan) (Liu et al., 2019; Stratton et al., 2019).

### Statistical Analyses

All data were represented as the mean of independent specimens ± SEM and statistical analysis were performed using the GraphPad Prism software version 8.0 (GraphPad Software, Inc., San Diego, CA). The between-group differences were analyzed using unpaired two-tailed Student’s *t*-test, for comparison among three or more groups. One or two-way analysis of variance (ANOVA) followed by post hoc Bonferroni–Dunn test comparison was applied for multiple groups. A p-value of ≤0.05 was considered statistically significant.

## Results

### Changes of Echocardiographic Properties and Myocardial Fibrosis in HFD-Induced T2DM Mice

**FIGURE 2:**
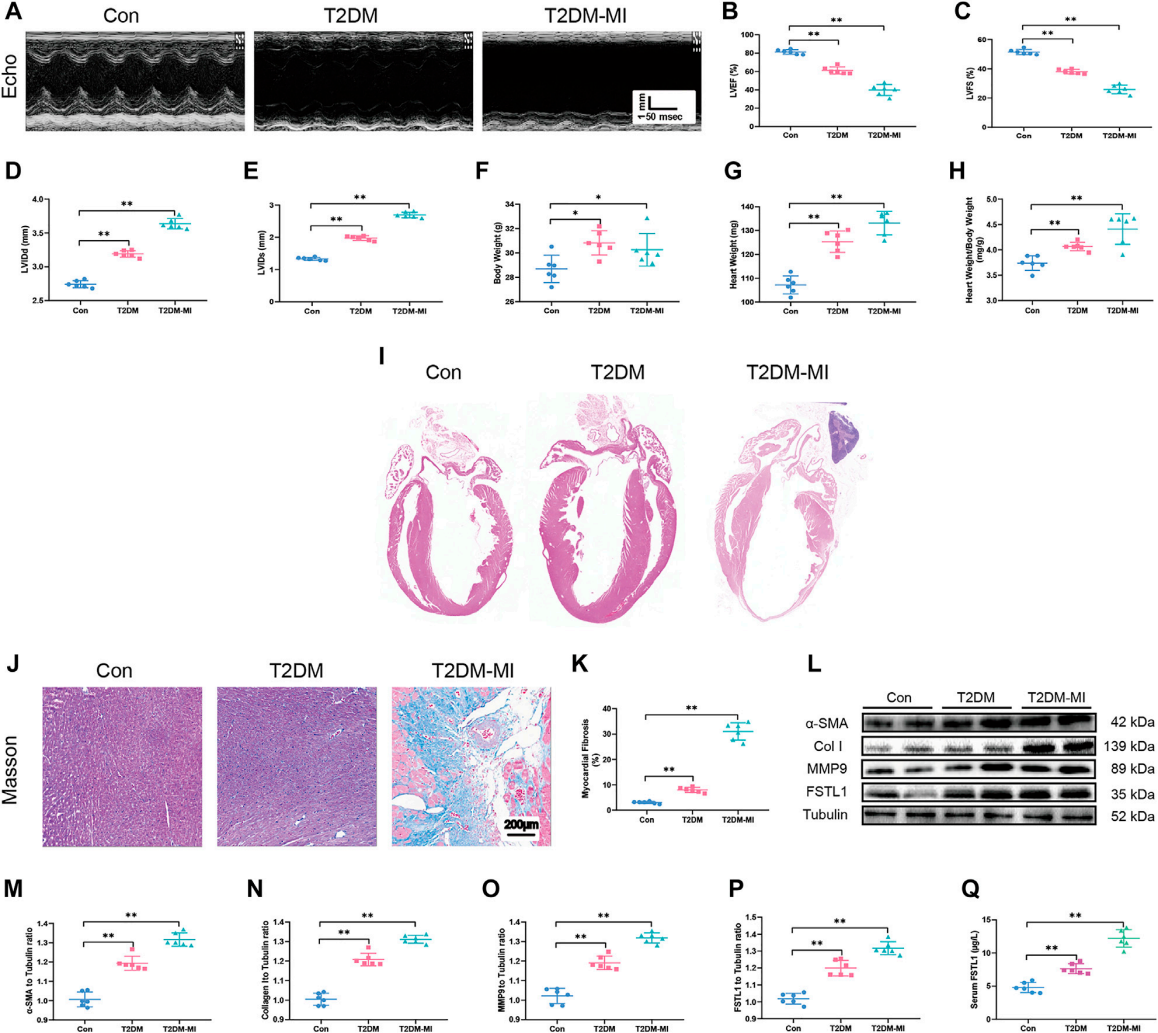
Echocardiographic function, levels of FSTL1 and myocardial fibrosis in high fat diet-induced T2DM mice following with sham or MI injury. **(A)** Representative M-mode images of echocardiography challenged with MI injury in T2DM mice; **(B)** Left ventricular ejection fraction (LVEF); **(C)** Left ventricular fractional shortening (LVFS); **(D)** left ventricular internal diameter at end-diastole (LVIDd); **(E)** Left ventricular internal diameter at end-systole (LVIDs); **(F)** Body weight; **(G)** Heart weight; **(H)** Heart weight to body weight ratio; **(I)** H&E staining; **(J)** Cardiac interstitial fibrosis using Masson’s Trichrome staining; **(K)** Pooled data of interstitial fibrotic area of myocardial tissues; **(L)** Representative images of Western blot in high fat diet-induced T2DM challenged with MI injury; **(M)** α-SMA level; **(N)** Collagen I level; **(O)** MMP9 levels; **(P)** FSTL1 levels; and **(Q)** Serum LDH level. Con: normal control; T2DM: high fat diet-induced T2DM; T2DM-MI, mice underwent myocardial injury in T2DM. Mean ± SEM, *n* = 6 per group. **p < 0.01. scale bar = 200 μM.

To discern echocardiographic dysfunction in mice with T2DM-associated MI, HFD-induced DM mice were used. Our results displayed in Figures 2A–C reveal that LVEF and LVFS were significantly decreased in the HFD-induced T2DM mice compared with those of the control group. LVEF and LVFS were further reduced in diabetic mice post-MI. LVIDd and LVIDs were slightly increased in diabetic mice (Figures 2D–E), along with increased body weight, heart weight, and heart weight to body weight ratio (Figures 2F–H), The above parameters, excepting body weight were further increased in T2DM mice post-MI.

**FIGURE 3:**
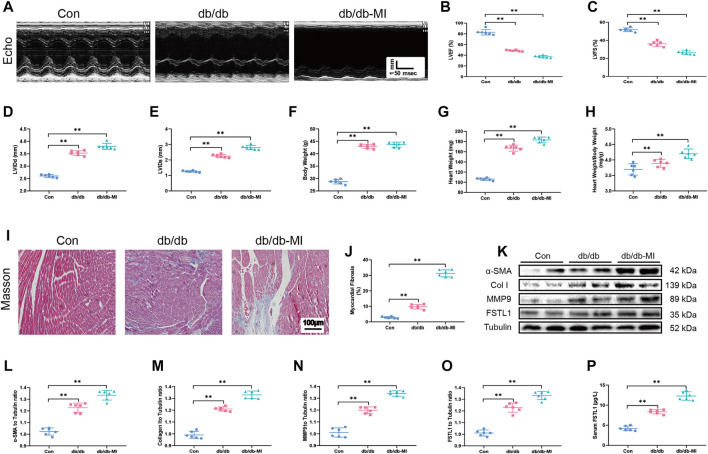
Echocardiographic function, levels of FSTL1 and myocardial fibrosis in diabetic db/db mice following with sham or MI injury. **(A)** Representative M-mode images of echocardiography challenged with MI injury in diabetic db/db mice; **(B)** Left ventricular ejection fraction (LVEF); **(C)** Left ventricular fractional shortening (LVFS); **(D)** left ventricular internal diameter at end-diastole (LVIDd); **(E)** Left ventricular internal diameter at end-systole (LVIDs); **(F)** Body weight; **(G)** Heart weight; **(H)** Heart weight to body weight ratio; **(I)** Cardiac interstitial fibrosis using Masson’s Trichrome staining; **(J)** Pooled data of the interstitial fibrotic area of myocardial tissues; **(K)** Representative images of Western blot in high fat diet-induced T2DM challenged with sham or MI injury; **(L)** α-SMA level; **(M)** Collagen I level; **(N)** MMP9 level; **(O)** FSTL1 level; and **(P)** Serum LDH level. Con: normal control; *db/db*: diabetic *db/db* mice; *db/db*-MI, *db/db* mice underwent myocardial injury. The results are presented as Mean ± SEM, n = 6 in each group. **p < 0.01. scale bar = 100 μM.

Our data revealed overtly increased myocardial fibrosis in T2DM mice with higher collagen deposition compared with control group. Elevated expressions of the myofibroblast marker, α-SMA (alpha-smooth muscle actin) and the fibrosis markers including MMP9 and collagen type I (collagen I), were observed in T2DM mice that underwent MI surgery which exhibited significantly increased myocardial fibrosis compared to the T2DM-only group as shown in Figures 2J–O. Additionally, FSTL1 were markedly increased in the cardiac tissues of T2DM mice and further increased in T2DM mice that underwent MI surgery (Figures 2L,P,Q and Figures 3K,O,P).

### Changes of Echocardiographic Properties and Myocardial Fibrosis in *Db/db* Mice

To detect echocardiographic dysfunction in mice with T2DM-associated MI, *db/db* mice were used. Our results depicted in Figures 3A–C demonstrated that LVEF and LVFS were significantly decreased in *db/db* mice compared with those in the control group and were further decreased post-MI in *db/db* mice. Additionally, LVIDd, LVIDs, heart weight, and heart weight to body weight ratio were significantly increased in T2DM post-MI compared to *db/db* mice, and diabetes alone dramatically increased these indicators (Figures 3D–H). Moreover, the cardiac interstitial fibrotic area evaluated by Masson’s trichrome staining and the expressions of myofibroblast marker α-SMA and the fibrotic markers including MMP9 and collagen type I were significantly increased in *db/db* mice and exhibited further elevation post-MI in *db/db* mice (Figures 3I–N). FSTL1 level was markedly increased in the myocardial tissues of *db/db* mice and further elevated by MI surgery (Figures 3O,P).

### Effect of FSTL1 on Cardiac Function, Myocardial Fibrosis and USP10 Levels in Post-MI T2DM Mice

**FIGURE 4:**
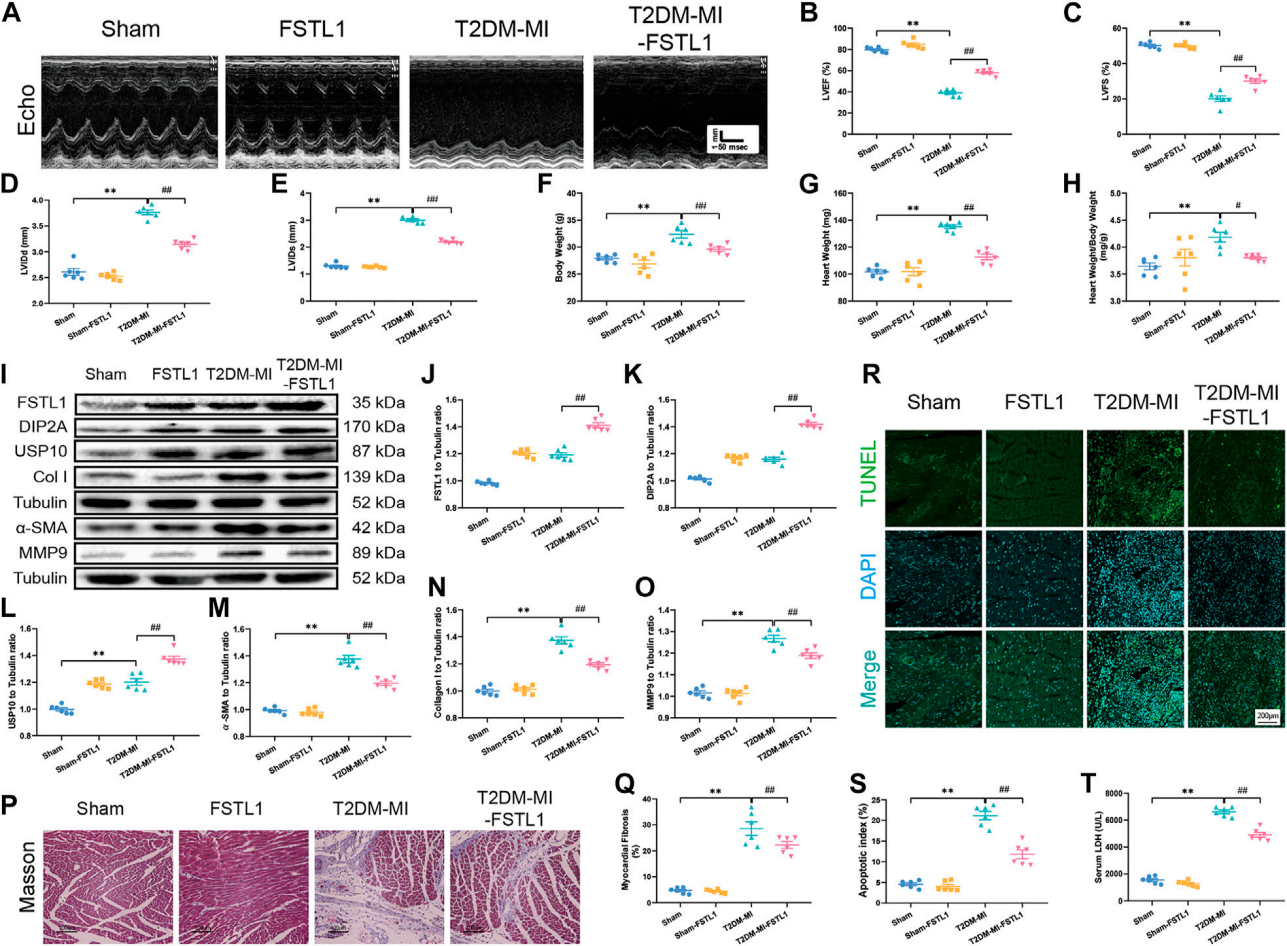
The effect of FSTL1 treatment on heart function, myocardial fibrosis, apoptotic index, and serum LDH level in T2DM mice following MI injury. **(A)** Representative M-mode images of echocardiography challenged with MI injury in T2DM mice with/without FSTL1 treatment; **(B)** Left ventricular ejection fraction (LVEF); **(C)** Left ventricular fractional shortening (LVFS); **(D)** Left ventricular internal diameter at end-diastole (LVIDd); **(E)** Left ventricular internal diameter at end-systole (LVIDs); **(F)** Body weight; **(G)** Heart weight; **(H)** Heart weight to body weight ratio; **(I)** Representative images of Western blot in T2DM mice challenged with sham or MI injury with/without FSTL1 treatment; **(J)** FSTL1 level; **(K)** DIP2A level; **(L)** USP10 level; **(M)** α-SMA level; **(N)** Collagen I level; **(O)** MMP9 level; **(P)** Cardiac interstitial fibrosis using Masson’s Trichrome staining; **(Q)** Pooled data of the interstitial fibrotic area of myocardial tissues; **(R)** Representative images of apoptotic cardiomyocytes. TUNEL: green fluorescence represents TUNEL positive nuclei; DAPI: blue fluorescence represents total cardiomyocyte nuclei; **(S)** Myocardial apoptosis was presented as the apoptotic index (×100%); and **(T)** Serum LDH level. Sham: mice were undergoing a sham operation; FSTL1: mice were undergoing a sham operation and intracardiac injected with AAV9-FSTL1; T2DM-MI: HFD induced T2DM mice underwent MI surgery and intracardiac injected with AAV9-NC; T2DM-MI-FSTL1: HFD induced T2DM mice underwent MI surgery and intracardiac injected with AAV9-FSTL1. The results are presented as Mean ± SEM, n = 6 in each group. **p < 0.01. Scale bar in **(Q)** was 100 μm and the scale bar in **(R)** was 200 μm.

Echocardiographic results indicated that LVEF and LVFS were overtly decreased along with significantly increased LVIDd, LVIDs, body weight, heart weight and heart weight to body weight in post-MI T2DM mice. MI further resulted in the elevation of myocardial apoptosis and serum LDH levels. Moreover, MI promoted myocardial fibrosis dramatically, by increasing collagen deposition and upregulated the expression of fibrotic markers including MMP9, collagen type I, and α-SMA, the effects of which were alleviated by FSTL1 administration (Figures 4A–H). Compared with the T2DM-MI group, FSTL1 treatment significantly improved cardiac function and alleviated MI-induced myocardial apoptosis as well as serum LDH levels in T2DM mice. However, FSTL1 treatment alone did not elicit any effect on cardiac function, myocardial fibrosis, apoptosis, or serum LDH levels compared to sham group (Figure 4I–T). Additionally, USP10 was significantly increased in T2DM mice that underwent MI surgery, which was further up-regulated by FSTL1 treatment (Figures 4I,L), suggesting that USP10 may play a pivotal role in MI- induced injury under T2DM.

### USP10 Inhibition Counteracted the Protective Role of FSTL1 and Might be Related to Notch1 Signaling in Diabetes Mellitus Accompanying MI Surgery

**FIGURE 5:**
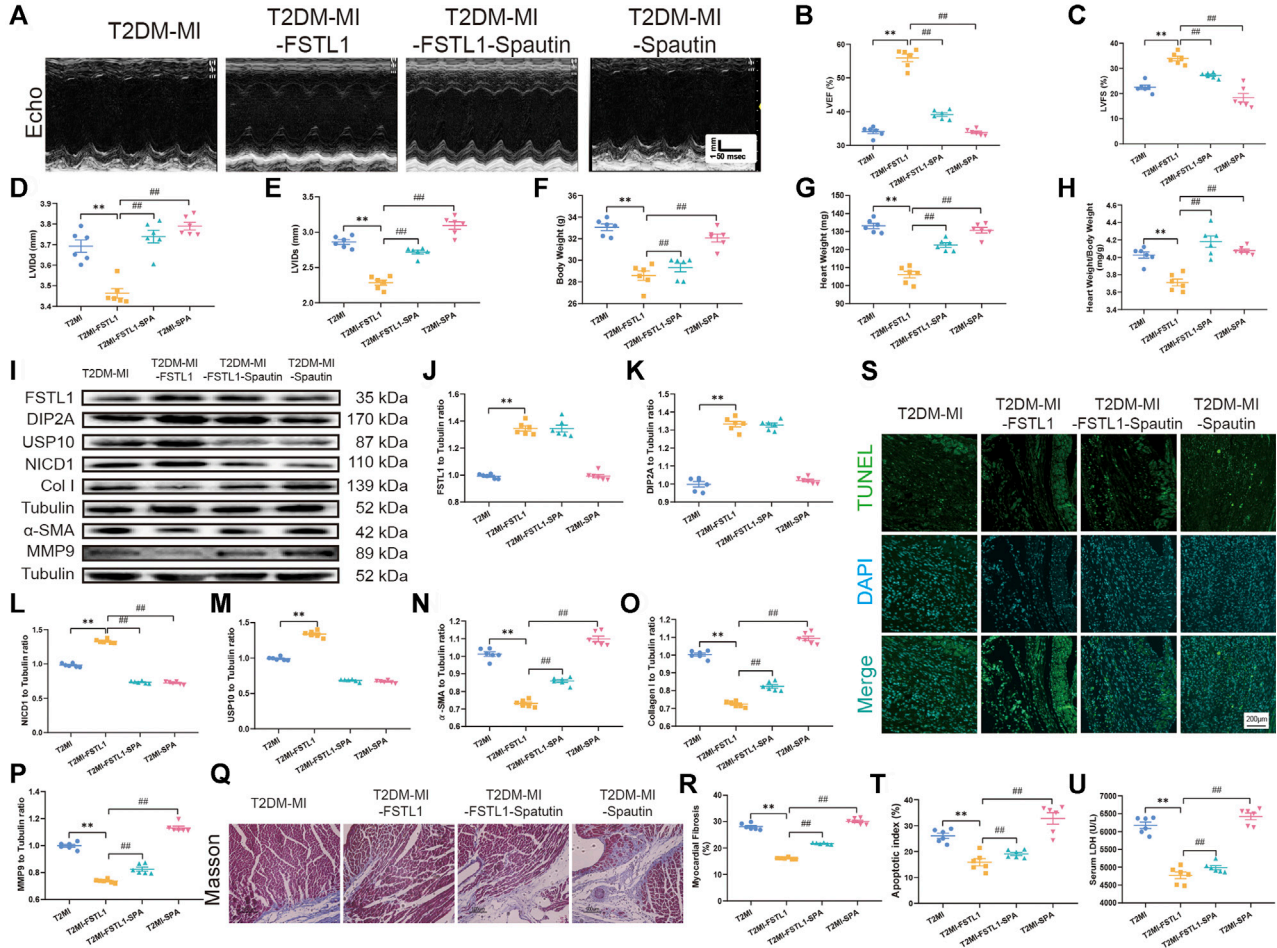
Echocardiographic function, levels of FSTL1 and myocardial fibrosis in T2DM post-USP10 inhibition. **(A)** Representative M-mode images of echocardiography challenged with MI injury in T2DM mice with/without FSTL1 treatment; **(B)** Left ventricular ejection fraction (LVEF); **(C)** Left ventricular fractional shortening (LVFS); **(D)** Left ventricular internal diameter at end-diastole (LVIDd); **(E)** Left ventricular internal diameter at end-systole (LVIDs); **(F)** Body weight; **(G)** Heart weight; **(H)** Heart weight to body weight ratio; **(I)** Representative images of Western blot in T2DM mice challenged with sham or MI injury with/without FSTL1 treatment; **(J)** FSTL1 level; **(K)** DIP2A level; **(L)** NICD1 level; **(M)** USP10 level; **(N)** α-SMA level; **(O)** Collagen I level; **(P)** MMP9 level; **(Q)** Cardiac interstitial fibrosis using Masson’s Trichrome staining; **(R)** Pooled data of the interstitial fibrotic area of myocardial tissues; **(S)** Representative images of apoptotic cardiomyocytes; TUNEL: green fluorescence represents TUNEL positive nuclei; DAPI: blue fluorescence represents total cardiomyocyte nuclei; **(T)** Myocardial apoptosis was presented as the apoptotic index (×100%); and **(U)** Serum LDH level. The results are presented as Mean ± SEM, *n* = 6 in each group. **p < 0.01. Scale bar in **(Q)** was 100 μm and the scale bar in **(R)** was 200 μm.

To evaluate the effect of USP10 on cardiac function, T2DM mice were treated with the USP10 selective inhibitor spautin-1 intraperitoneally for 4 weeks post MI. Echocardiographic data demonstrated that FSTL1 administration significantly increased LVEF and LVFS and decreased LVIDd, LVIDs, heart weight and heart weight to body weight ratio compared to those in T2DM-MI group. USP10 inhibition by spautin-1 significantly offset the protective role of FSTL1 in T2DM mice that underwent MI (Figures 5A–H). Spautin-1 treatment aggravated myocardial fibrosis by increasing collagen deposition and upregulated the expression of the fibrotic markers including MMP9, collagen type I, and α-SMA, or promoted myocardial apoptosis and increased serum LDH levels, which neutralized the protective effect of FSTL1 in T2DM mice that underwent MI surgery. It was noteworthy that spautin-1 treatment alone promoted cardiac dysfunction and increased myocardial fibrosis and apoptosis in T2DM mice following MI surgery (Figures 5I–U). Simultaneously, the data in Figure 5 revealed that the expression of Notch1 and USP10 were elevated in cardiac tissue post MI in T2DM, and further activated by FSTL1 administration. Nevertheless, that response was nullified by spautin-1 treatment.

### Notch1 Inhibition Reduced the Myocardial Protective Effect of FSTL1 Challenged With High Glucose/High Fat and Hypoxia-Induced Injury but Did Not Affect the Activation of USP10 *in Vitro*

**FIGURE 6:**
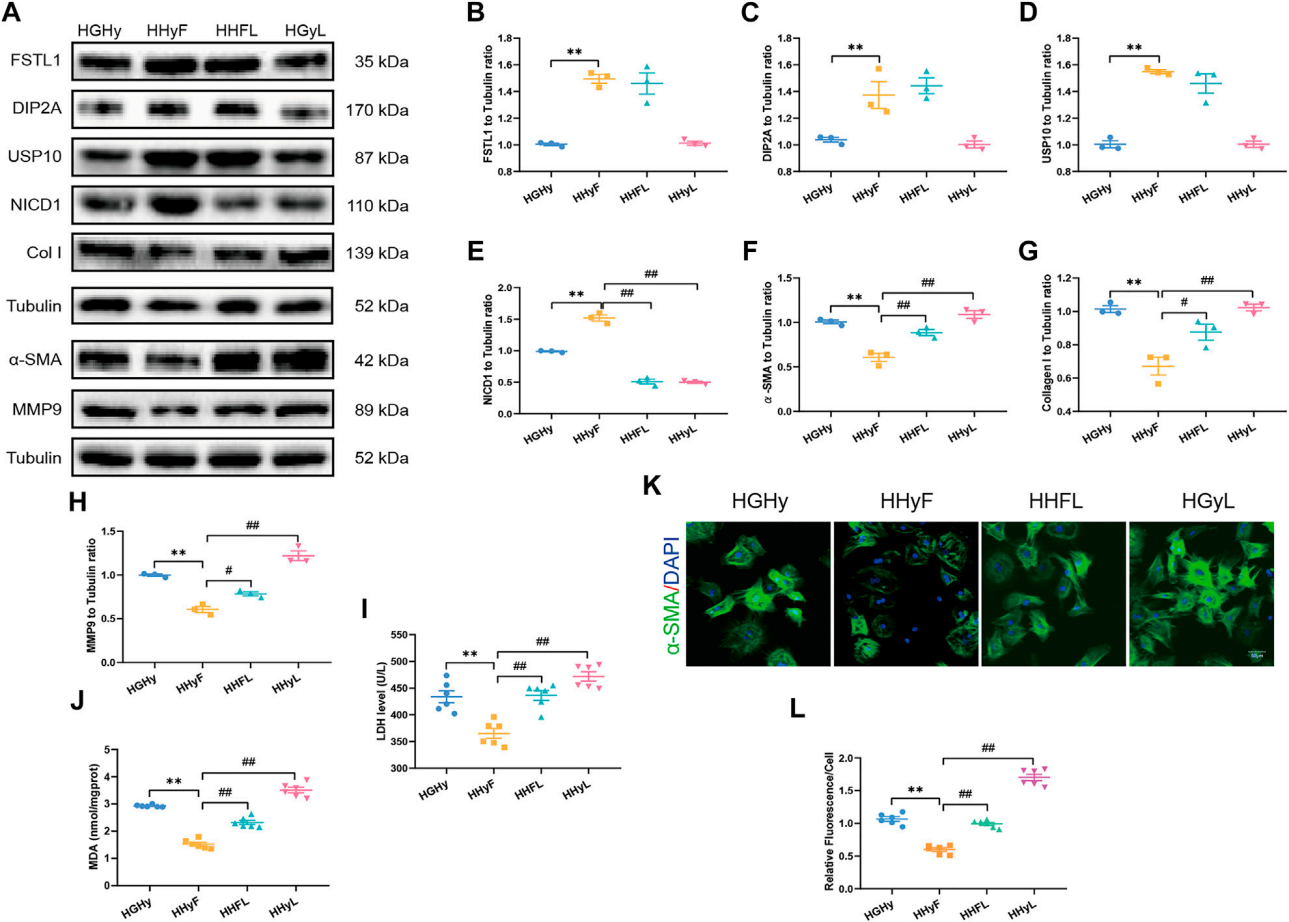
Levels of FSTL1, UPS10 and myocardial fibrosis related proteins in CFs isolated from T2DM mice post-notch1 inhibition. **(A)** Representative images of Western blot in CFs isolated from T2DM; **(B)** FSTL1 level; **(C)** DIP2A level; **(D)** USP10 level; **(E)** NICD1 level; **(F)** α-SMA level; **(G)** Collagen I level; **(H)** MMP9 level; **(I)** LDH level; **(J)** MDA content; **(K)** Immunofluorescence staining of α-SMA; nuclei are marked by Hoechst 33342 staining; scale bar = 50 μm; Green fluorescence represents α-SMA positive nuclei; DAPI: blue fluorescence represents total CFs nuclei; and **(L)** Quantification of α-SMA staining. HGHy: isolated CFs were treated with dimethyl sulfoxide (DMSO) as control; HHyF: isolated CFs were treated with 50 ng/ml recombinant Fstl1 protein for 6 h; HHFL: isolated CFs were treated with crenigacestat (LY3039478, 10 μM) 18 h before recombinant Fstl1 protein administration, and then co-cultured for 6 h; HHyL: isolated CFs were treated with crenigacestat for 24 h. The results are presented as Mean ± SEM, *n* = 3–6 in each group. **p < 0.01.

Our data in Figures 6A–J demonstrated that FSTL1 treatment inhibited CF activation by down-regulating the expression of fibrotic markers including MMP9, collagen type I, and α-SMA. Meanwhile, LDH levels, and MDA content were decreased while the expressions of USP10 and Notch1 were upregulated in T2DM-MI-induced myocardial injury following FSTL1 treatment. The Notch1 inhibitor, crenigacestat (LY3039478) markedly abolished the inhibition of fibrotic marker expression by FSTL1 treatment but did not affect the protein level of USP10. In addition, crenigacestat treatment alone increased the expression of fibrotic markers including MMP9, collagen type I, and α-SMA but did not alter the USP10 protein levels. The inhibitory effect of FSTL1 on relative fluorescence of α-SMA was counteracted by crenigacestat (Figures 6K,L).

### USP10/Notch1 Signaling Pathway Participated in the Protective Role of FSTL1 in DM-MI-Induced CFs Injury

**FIGURE 7:**
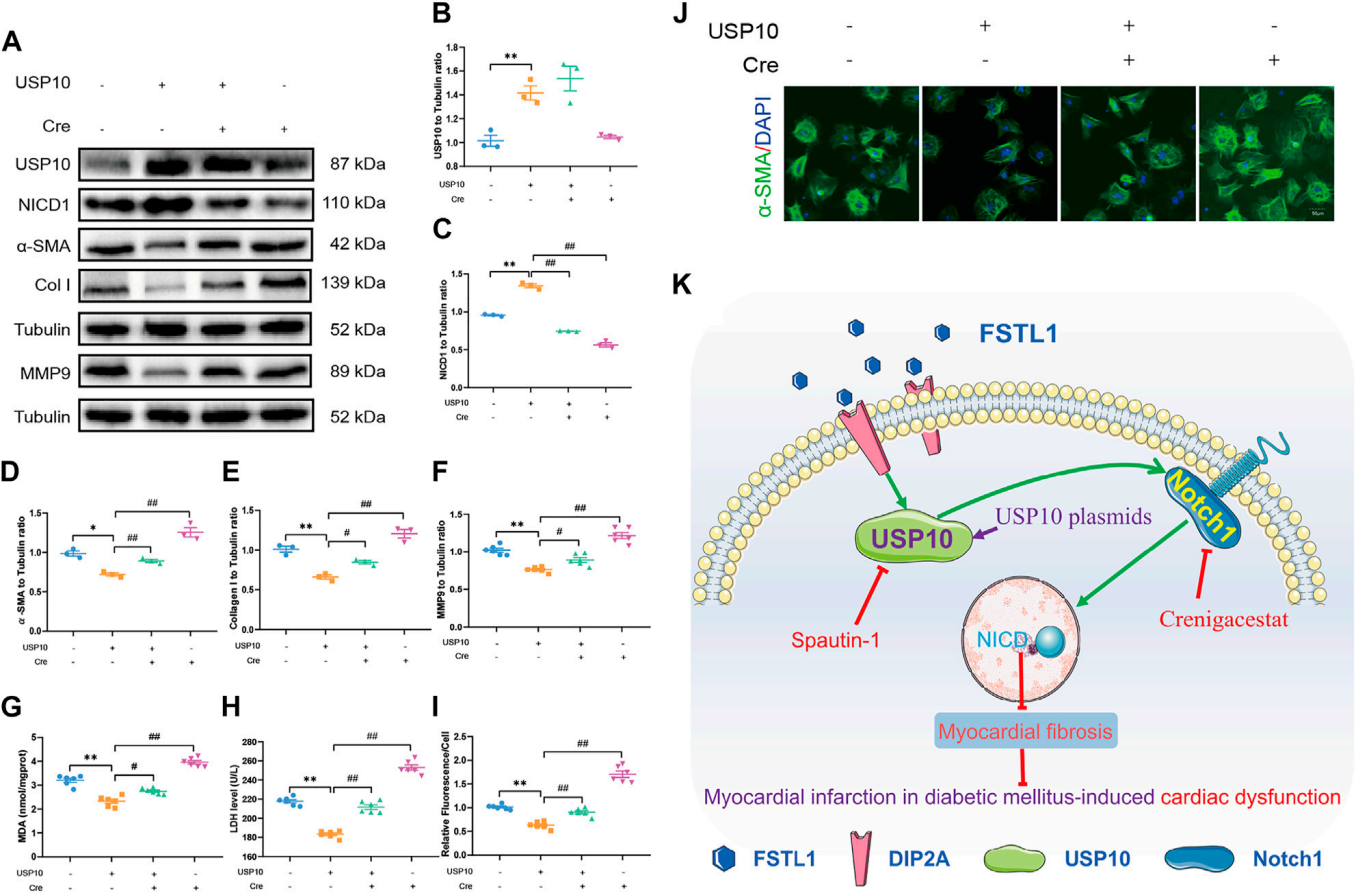
Levels of UPS10, NICD1 and myocardial fibrosis related proteins in CFs isolated from T2DM mice post-notch1 inhibition. **(A)** Representative images of Western blot in CFs with USP10 treatment; **(B)** USP10 level; **(C)** NICD1 level; **(D)** α-SMA level; **(E)** Collagen I level; **(F)** MMP9 level; **(G)** MDA content; **(H)** LDH level; **(I)** Quantification of α-SMA staining. **(J)** Immunofluorescence staining of α-SMA; nuclei are marked by Hoechst 33342 staining; scale bar = 50 μm; Green fluorescence represents α-SMA positive nuclei; DAPI: blue fluorescence represents total CFs nuclei; and K: FSTL1-USP10-notch1 signaling axis plays a critical role in attenuating myocardial infarction by inhibition myocardial fibrosis in diabetic mice. The results are presented as Mean ± SEM, *n* = 3–6 in each group. **p < 0.01.

To further elucidate the role of USP10/Notch1 signaling in CFs isolated from T2DM mice, CFs were treated with crenigacestat to inhibit Notch1 signaling while USP10 plasmids were employed to activate USP10. USP10 plasmid treatment enhanced the protective role of USP10 in CFs injury as evidenced by significantly reduced levels of fibrostic markers including MMP9, collagen type I, and α-SMA as well as LDH and MDA levels, in keeping with *in vivo* results. On the other hand, crenigacestat disengaged the protective effect of USP10 in CFs injury (Figures 7A–H). Treatment with USP10 plasmid also blunted the formation of α-SMA-positive stress fibers in CFs isolated from T2DM-MI mice. These findings reveal the role of Notch1 as a downstream signal for USP10 against MI-induced cardiac injury in T2DM.

## Discussion

Our present study revealed that FSTL1 protected cardiac function against MI-induced myocardial fibrosis in T2DM through a USP10/Notch1-dependent mechanism. Our *in vivo* study revealed that intracardiac delivery of AAV9-FSTL1 alleviated cardiac dysfunction by activating USP10 and alleviating myocardial fibrosis. Further *in vitro* analysis provided evidence that FSTL1 activated USP10/Notch1 signaling to inhibit myocardial fibrosis. Our combined data demonstrates a pivotal protective role of FSTL1 against MI injury in T2DM through a USP10/Notch1-dependent manner.

The coexistence of diabetes and heart failure pose unique clinical challenges because DM independently increases the risk of heart failure by 2–5-fold, and 44% of patients hospitalized for heart failure have DM (Ritchie & Abel 2020). Besides, a glycated hemoglobin level above the normal range was the strongest predictor of MI, with DM persisting as an independent predictor of poor outcome (Rawshani et al., 2018; Ritchie & Abel 2020). The delivery of oxygen and other essential nutrients to the myocardium were reduced by DM-induced microvascular rarefaction which has been identified as a predictor of cardiac fibrosis with a robust elevation in interstitial collagen types I and III (Ritchie & Abel 2020). Furthermore, as an important complication of diabetes, coronary artery disease such as MI further aggravates the occurrence of myocardial fibrosis (Dillmann 2019; Zelniker et al., 2019). As was reported that cardiac fibroblast activation has pivotal role against MI-induced cardiac rupture at an early stage (Maruyama et al., 2016) while cardiac stiffness was significantly increased with the continuous increase of myocardial fibrosis (Zhang et al., 2021). If there has a therapeutic strategy that can balance this pathological change? Previous studies have suggested that FSTL1 attenuated pathological injury of multiple organs by alleviating fibrosis, particularly in cardiac dysfunction (Jiang et al., 2020; Hu et al., 2020; Li et al., 2021; Z.; Chen Z. et al., 2019; Yang et al., 2020). Our current findings are consistent with these aforementioned research findings that the upregulation of FSTL1 in DM compared with the control group, which was further increased in T2DM with MI. Further studies must investigate the molecular mechanism of FSTL1 in myocardial fibrosis with T2DM post-MI.

It was previously confirmed that FSTL1 is associated with insulin resistance and that circulating FSTL1 levels were elevated in patients with T2DM. FSTL1 may be involved in the pathogenesis of fibrosis in diabetic retinopathy caused by anti-VEGF (vascular endothelial growth factor) treatment (Xu et al., 2020). FSTL1 protected the heart against I/R (ischemia-reperfusion) injury by reducing myocardial infarct size and improved cardiomyocyte survival by increasing the phosphorylation of Akt and ERK signaling (Oshima et al., 2008). Normalizing energy metabolism and AMPK-activated oxygen consumption are involved in the protection of cardiac diastolic and contractile function following an increase of serum FSTL1 (Seki et al., 2018). It was further confirmed that FSTL1 delivered through an epicardial patch protected the heart against MI injury by promoting cardiomyocyte division (Wei et al., 2015). Further, FSTL1 protected the heart against MI injury by decreasing myocardial fibrosis, and protected the heart from rupture by regulating cardiac fibroblast activation (Rainer et al., 2014; Maruyama et al., 2016; Xiao et al., 2019; Hu et al., 2020). Importantly, upregulation of FSTL1 secretion induced by exercise training alleviated cardiac dysfunction resulting from MI injury by TGFβ-Smad2/3 induced angiogenesis (Xi et al., 2016). Besides, it was reported that coronary artery disease such as MI further would aggravate the occurrence of myocardial fibrosis (Dillmann 2019; Zelniker et al., 2019). Nevertheless, the role of FSTL1 in T2DM coexisting with MI surgery has not yet been elucidated. Our *in vivo* study demonstrated that protein levels of Cleaved Caspase 3 and Bax were significantly decreased while Beclin 1 was increased following FSTL1 treatment (Supplementary Figure S5A). However, USP10 inhibition did not have any effect on Beclin 1 (Supplementary Figure S5B). The present findings indicate that intracardiac injection with AAV9-FSTL1 remarkably improved cardiac function and decreased myocardial apoptosis without altering the body weight of mice with T2DM post-MI. Moreover, cardiac dilation manifested by LVIDs and LVIDd was inhibited after intracardiac injection with AAV9-FSTL1.

The extracellular matrix (ECM) provides a scaffold for healthy cardiomyocytes to maintain the structural integrity and function of the heart. Cardiac remodeling could disrupt the proper excitation-contraction coupling and may promote arrhythmia, in which, myocardial ischemia and diabetes are important pathogenic factors (Jia et al., 2018; Bacmeister et al., 2019; Friebel et al., 2019; Nagaraju et al., 2019). Numerous studies have confirmed the association of expansion of the cardiac interstitium and collagen deposition with activation of a matrix-synthetic program in cardiac fibroblasts but not in myofibroblast conversion in mouse models, whereas prolonged ischemia induced by MI could trigger an inflammation-driven reparative fibrotic response and scar formation in the sub-epicardium. Larger scars are often associated with worse prognosis in patients (Frangogiannis 2020). Li et al. demonstrated myocardial fibrosis in diabetic mice and cardiac fibrosis in mice that underwent MI and in neonatal rat CFs was significantly increased as well (Li et al., 2019; Gao et al., 2020) with cardiac dysfunction and cardiac remodeling, but patients with diabetes confers a markedly increased risk of death and clinical MI (Elliott et al., 2019; Jia et al., 2019). Our present results indicate that diabetes increases myocardial fibrosis, and MI further aggravates the degree of myocardial fibrosis, which is consistent with our previous study (Lu et al., 2020b). The delivery of AAV9-FSTL1 significantly decreased myocardial fibrosis in T2DM mice that underwent MI. Numerous studies have confirmed that Notch1 upregulation inhibited myocardial fibrosis in animal MI models whereas, Notch1 inhibition worsened cardiac dysfunction by increasing myocardial fibrosis in both MI injured hearts and in diabetic heart (Russell et al., 2011; Yu et al., 2018; Zhou et al., 2019; Xuan et al., 2020). Our study showed that Notch1 signaling was inhibited in T2DM mice with MI and that cardiac fibrosis or cardiac dysfunction increased. FSTL1 treatment significantly reduced myocardial fibrosis and improved cardiac function by activating the Notch1 signaling pathway, which was reversed by Notch1 inhibitor. Further investigation was focused on how FSTL1 regulates Notch activity following MI surgery in T2DM mice.

Previous studies established the cross-talk between USP10 and fibrosis in multiple tissues, which are down-regulated in cancers, Parkinson disease (PD), and keloids, and specifically render the heart susceptible to hypertrophy injury (Anisimov et al., 2019; Deng et al., 2019; Boumil et al., 2020; Zhang et al., 2020). These studies provided compelling evidence confirming the role of USP10 in cardiac hypertrophy, and indicated that USP10 inhibition exacerbated pressure overload-induced cardiac dysfunction and facilitated angiotensin II-induced cardiomyocyte injury. In addition, myocardial fibrosis in perivascular and interstitial spaces were significantly increased in USP10-CKO mice (Zhang et al., 2020). Research on the endothelium revealed that USP10, as a regulatory protein, delayed the rate of NICD1 degradation by deubiquitinating the Notch1 receptor (Lim et al., 2019). Consistent with the above findings, we further examined the changes in FSTL1 and USP10 in T2DM with and without MI and investigated the relationship between FSTL1 and USP10/Notch1 signaling. The present study revealed that FSTL1 and USP10 were significantly activated in T2DM mice with MI, and FSTL1 treatment further increased USP10 activation and alleviating cardiac dysfunction by reducing myocardial fibrosis. Conversely, USP10 inhibition significantly offset the cardioprotective effect of FSTL1 by increasing myocardial fibrosis. *In vitro* analysis confirmed that USP10 regulated the physiological function of CFs through the Notch signaling pathway. The protective effect of FSTL1 on CFs was counteracted following treatment with Notch1 inhibitor, whereas the activity of USP10 was not been affected. Moreover, Notch1 inhibitor treatment blocked the protective effect of USP10 in CFs injury, suggesting that Notch1, as a downstream key molecule of USP10, was involved in the protective effect exerted by FSTL1 on MI in T2DM.

## Conclusion

In conclusion, our results revealed a novel mechanism by which USP10-mediated activation of Notch1 plays a protective role against MI injury in T2DM mice. Specifically, the pivotal role of FSTL1 appears critical in cardiac protection. This study expands the current knowledge of the protective effects of FSTL1 in cardiovascular disease and may have potential clinical relevance against MI injury in diabetes. Moreover, our results establish a crosstalk between USP10 and Notch1 in the inhibition of myocardial fibrosis and improvement in cardiac function by alleviating adverse remodeling against MI injury in T2DM and may provide better prognosis for T2DM patients with MI. The basic knowledge reported in the present study provides a strategy that treatment with FSTL1 could be a promising pharmacological intervention for attenuating MI in T2DM and function and identifies USP10-notch1 signaling pathway as a novel factor that could contribute to the prevention of MI in T2DM.

## Data Availability

The original contributions presented in the study are included in the article/Supplementary Material, further inquiries can be directed to the corresponding authors.
