# Supplementary material for: FSTL1-USP10-Notch1 Signaling Axis Protects Against Cardiac Dysfunction Through Inhibition of Myocardial Fibrosis in Diabetic Mice
Source: Front Cell Dev Biol. 2021 Dec 9;9:757068. doi: 10.3389/fcell.2021.757068 (PMC8695978; doi:10.3389/fcell.2021.757068)
Supplement: Supplementary file 1 [file DataSheet1.docx]

**Expanded Methods**

Determination of Blood Glucose Levels.

A blood glucometer (Accu-Chek^®^ Active, Roche Diagnostics, Mannheim, Germany) was used for fasting plasma glucose levels determination as previously described (Lu et al., 2020). Briefly, mouse blood was obtained from the tail vein 3 days after STZ injection, mice with fasting plasma glucose >11.1 mmol/L were confirmed for type 2 diabetes mellitus.

Myocardial infarct size determination

To evaluate the infarct size between T2DM and T2DM-MI mice after MI, heart were harvested and frozen at -80℃. Then the cardiac tissue was cut transversely into 5 pieces and stained with 1% 2,3,5-triphenyltetrazolium chloride (TTC) solution (pH 7.4) for 30 minutes at 37°C. The slices were photographed after fixation for 4-6 hours in 10% neutral buffered formaldehyde and then infarct area were measured by using Image J software. Viable myocardium stained brick red, and infarct tissues appeared pale white. The infarct size was expressed as infarct area/total LV area ×100%.

Myocardial infarction biomarker determination

Blood samples were obtained at the end of the experiment and stored in a non-anticoagulant tube at 25℃ for 30min. The samples were then centrifuged at 3000 × g for 10 min and the serum was collected for the determination of creatine kinase MB (CK-MB, Nanjing Jiancheng, China) and cardiac troponin I (cTnI, Elabscience Biotechnology Co., Ltd., Wuhan) according to the manufactures’ instruction.

**Supplementary Figure Legends**


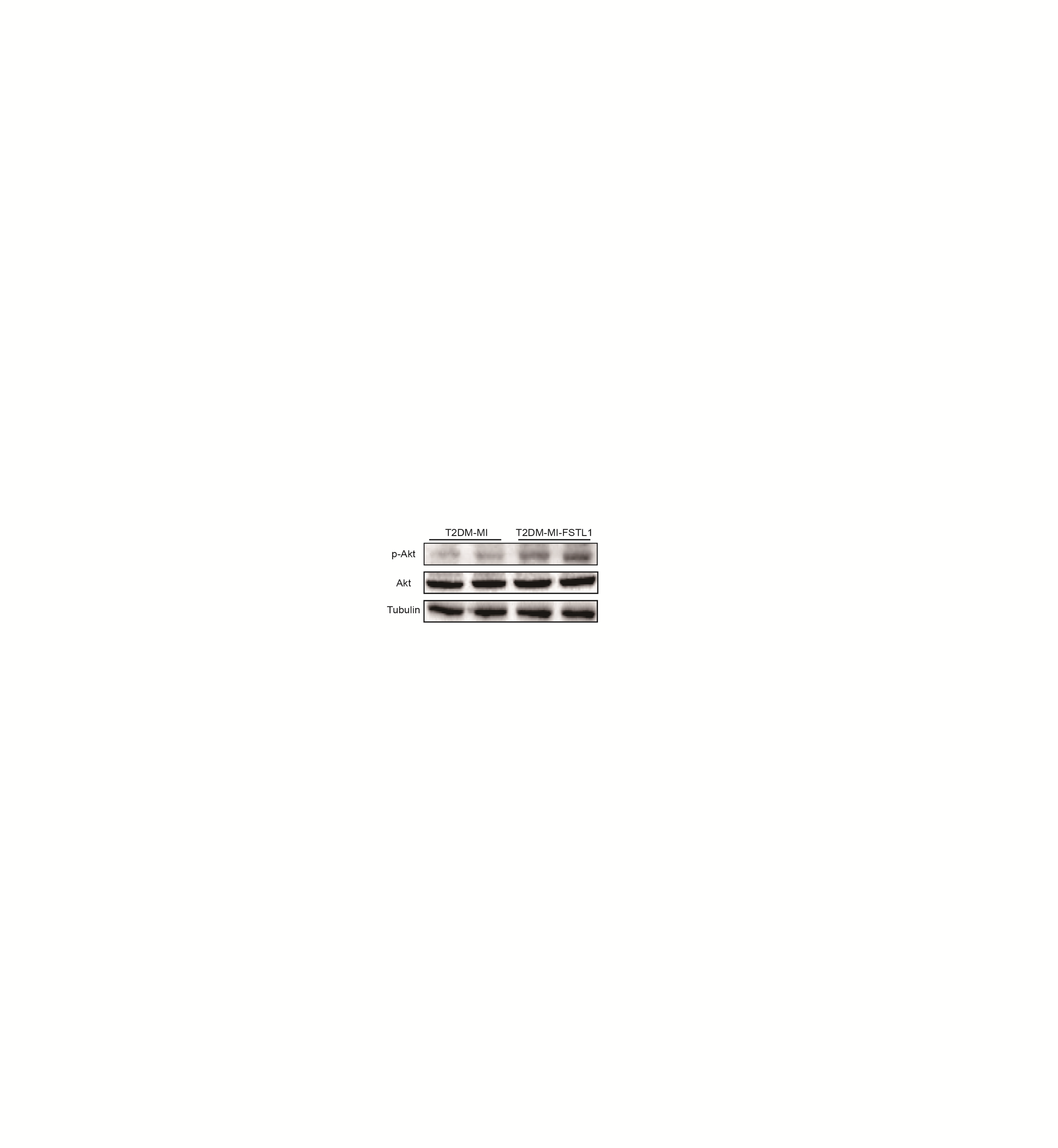


Figure S1. p-Akt was upregulated with FSTL1 delivered by the AAV9-FSTL1 vector in high fat diet-induced type 2 diabetes mellitus mice following MI injury.


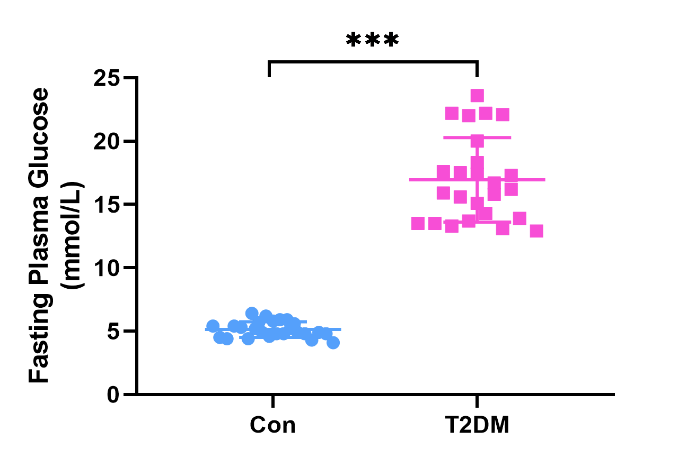


Figure S2. Fasting plasma glucose measurement in WT/T2DM mice, dot plot showing quantification of fasting plasma glucose in the normal control group and type 2 diabetes mellitus group. n = 20 in each group. ***P<0.001.


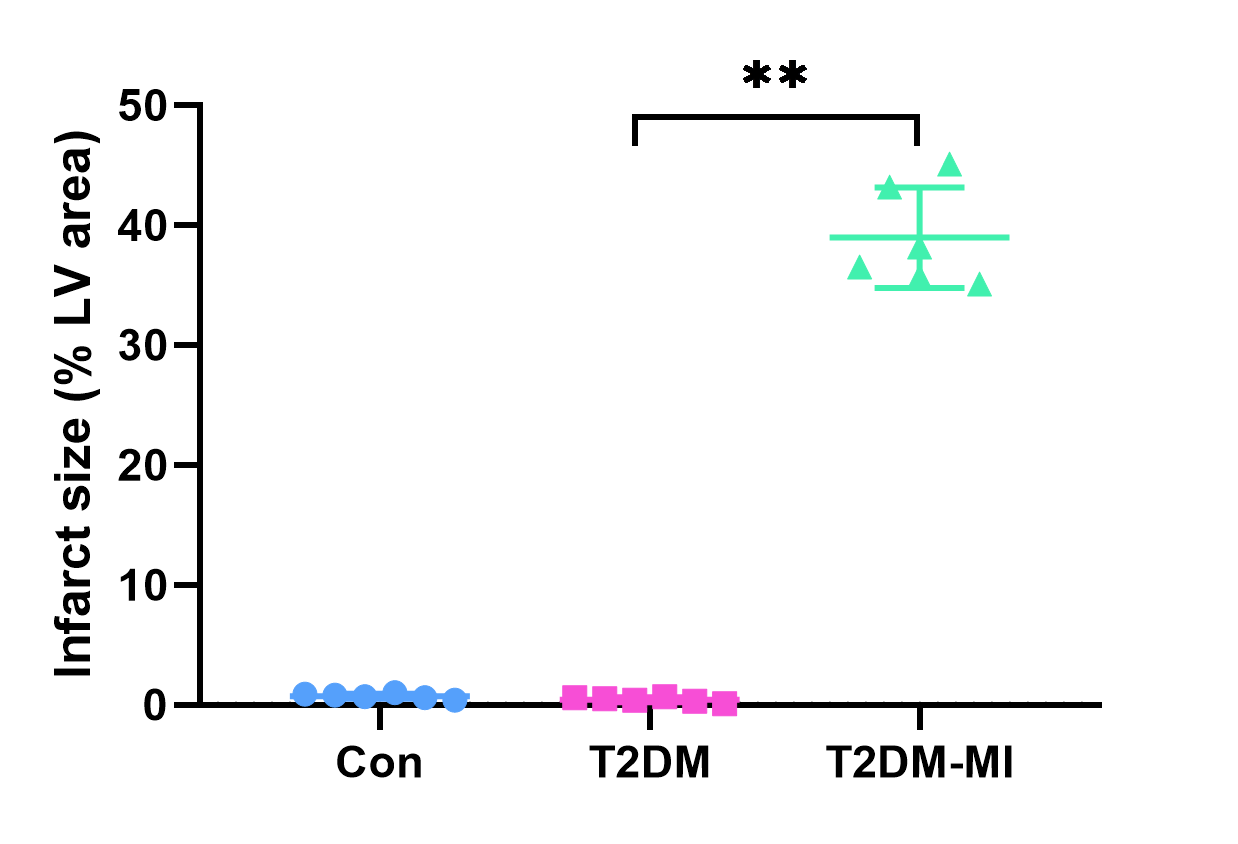


Figure S3. Myocardial infarct size measurement in WT/T2DM mice subjected to MI, dot plot showing quantification of infarct size in Con group, T2DM group, and T2DM-MI group. Con: normal control; T2DM: high-fat diet-induced T2DM; T2DM-MI, mice underwent myocardial injury in T2DM. n = 6 in each group. **P<0.01.


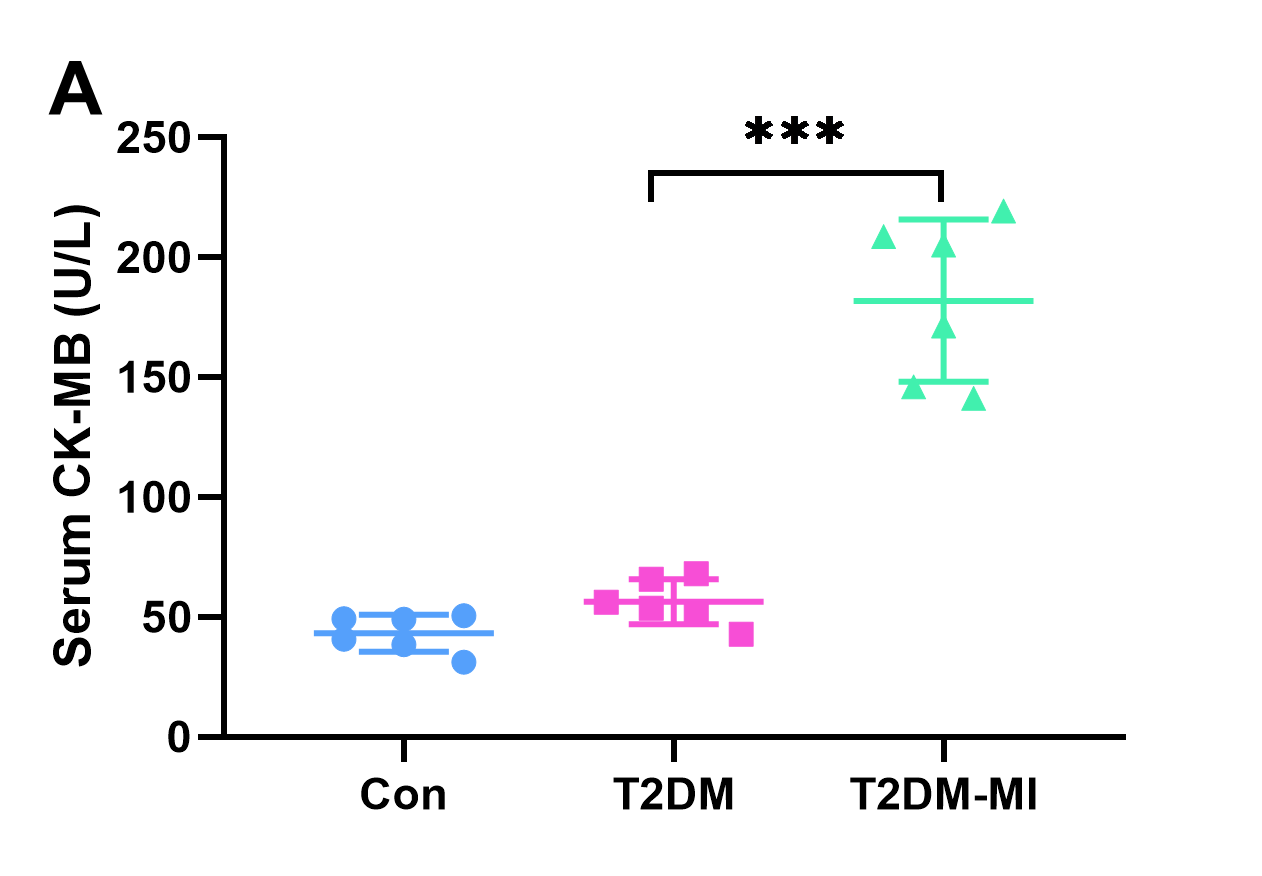

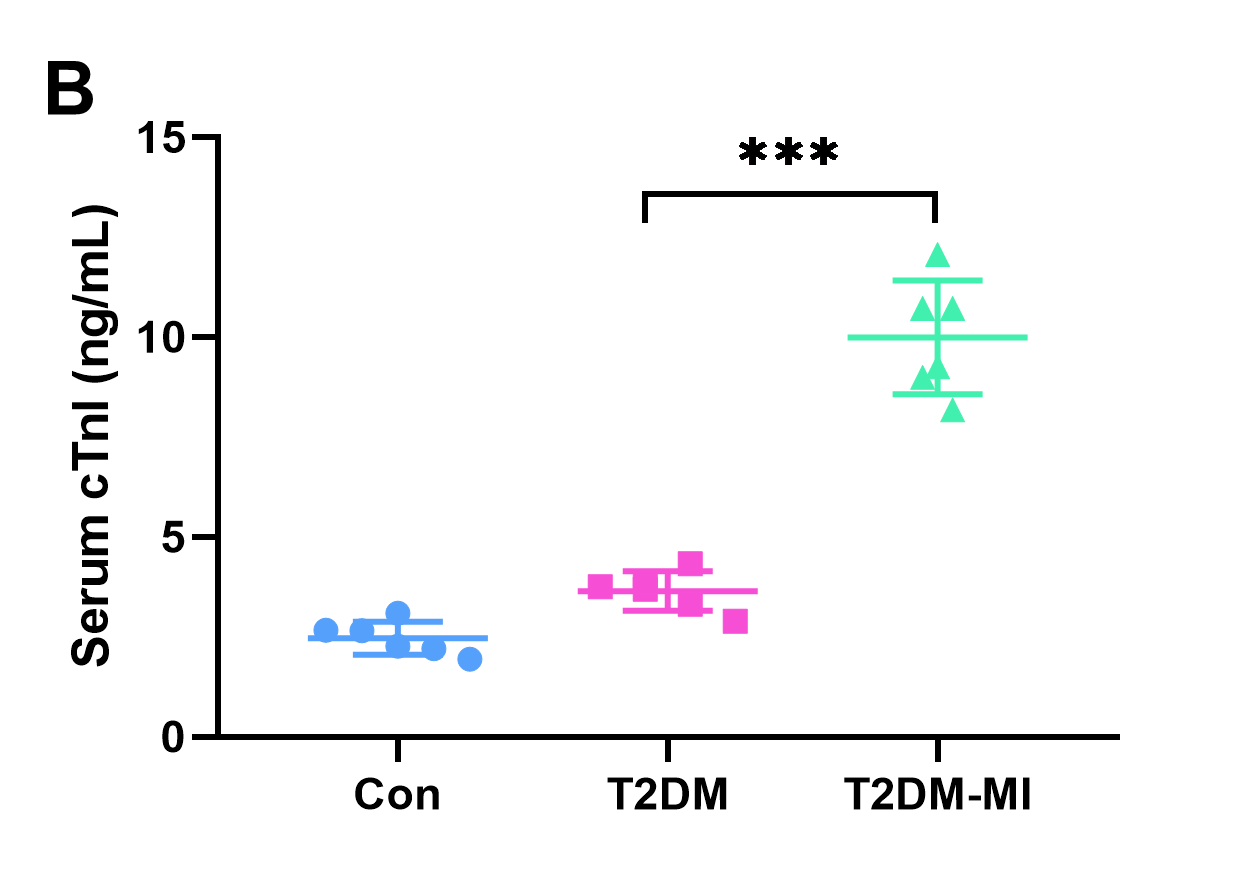


Figure S4. Serum CK-MB and cTnI levels in WT/T2DM mice subjected to MI. A, serum CK-MB level. B, serum cTnI level. Con: normal control; T2DM: high-fat diet-induced T2DM; T2DM-MI, mice underwent myocardial injury in T2DM. n = 6 in each group. ***P<0.001.


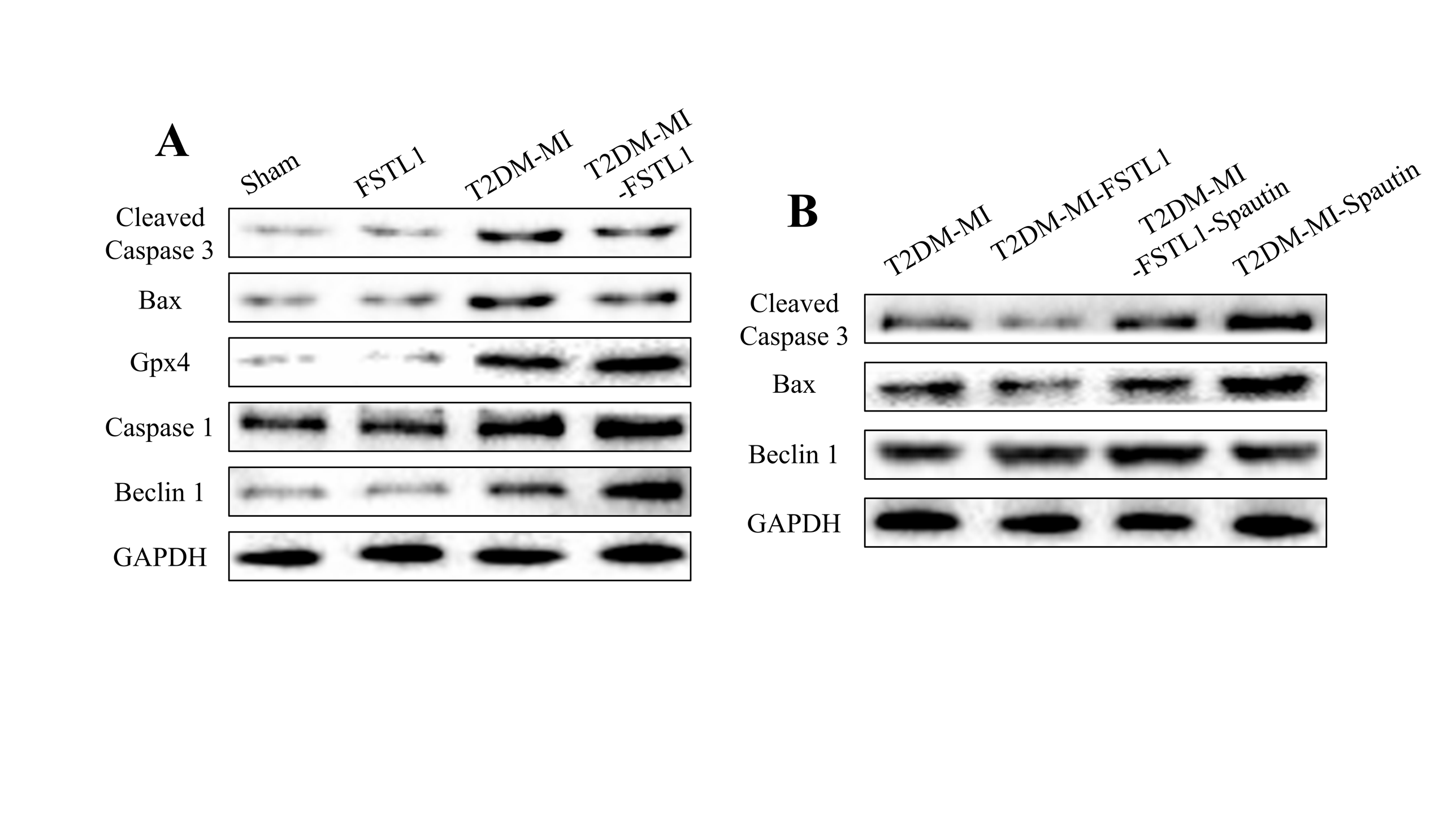


Figure S5. Cell death detection in WT/T2DM mice subjected to MI. A, the effect of FSTL1 treatment on apoptotic (cleaved caspase 3, Bax), ferroptosis (Gpx4), pyroptosis (Caspase 1), and autophagy (Beclin 1) in T2DM mice subjected to MI. B, the biomarkers of apoptotic and autophagy in T2DM-MI post-USP10 inhibition.

**References**

L. Lu, J. Ma, M. Sun, X. Wang, E. Gao, L. Lu, et al.(2020). Melatonin Ameliorates MI-Induced Cardiac Remodeling and Apoptosis through a JNK/p53-Dependent Mechanism in Diabetes Mellitus. *Oxid Med Cell Longev*. 2020, 1535201. doi:10.1155/2020/1535201
